# Effects of the ACT OUT! Social Issue Theater Program on Social-Emotional Competence and Bullying in Youth and Adolescents: Protocol for a Cluster Randomized Controlled Trial

**DOI:** 10.2196/17900

**Published:** 2020-04-13

**Authors:** Jon Agley, Wasantha Jayawardene, Mikyoung Jun, Daniel L Agley, Ruth Gassman, Steve Sussman, Yunyu Xiao, Stephanie L Dickinson

**Affiliations:** 1 Prevention Insights Department of Applied Health Science School of Public Health, Indiana University Bloomington Bloomington, IN United States; 2 Departments of Preventive Medicine and Psychology, and School of Social Work University of Southern California Los Angeles, CA United States; 3 Silver School of Social Work New York University New York, NY United States; 4 Biostatistics Consulting Center School of Public Health, Indiana University Bloomington Bloomington, IN United States

**Keywords:** social and emotional learning, bullying, social-emotional competence, psychodrama, randomized controlled trial

## Abstract

**Background:**

Students in the United States spend a meaningful portion of their developmental lives in school. In recent years, researchers and educators have begun to focus explicitly on social and emotional learning (SEL) in the school setting. Initial evidence from meta-analyses suggests that curricula designed to promote SEL likely produce benefits in terms of social-emotional competence (SEC) and numerous related behavioral and affective outcomes. At the same time, there are often barriers to implementing such curricula as intended, and some researchers have questioned the strength of the evaluation data from SEL programs. As part of the effort to improve programming in SEL, this paper describes the protocol for a cluster randomized trial of the ACT OUT! Social Issue Theater program, a brief psychodramatic intervention to build SEC and reduce bullying behavior in students.

**Objective:**

The objective of this trial is to examine if a short dose of interactive psychodrama can affect SEC metrics and bullying experiences in schoolchildren in either the short (2-week) or medium (6-month) term.

**Methods:**

The ACT OUT! trial is a cluster randomized superiority trial with 2 parallel groups. The unit of measurement is the student, and the unit of randomization is the classroom. For each grade (fourth, seventh, and 10th), an even number of classrooms will be selected from each school—half will be assigned to the intervention arm and half will be assigned to the control arm. The intervention will consist of 3 moderated psychodramatic performances by trained actors, and the control condition will be the usual school day. Outcome data will be collected at baseline (preintervention), 2-week postintervention (short term), and 6-month postintervention (medium term). Outcomes will include social-emotional competency; self-reported bullying and experiences of being bullied; receptivity to the program; and school-level data on truancy, absenteeism, and referrals to school displinary action for bullying. A power analysis adjusted for clustering effect, design effect, and potential attrition yielded a need for approximately 1594 students, consisting of an estimated 80 classrooms split evenly into intervention and control arms.

**Results:**

This study was funded in June 2019; approved by the Indiana University Institutional review board on September 17, 2019; began subject recruitment on November 5, 2019; and prospectively registered with ClinicalTrials.gov.

**Conclusions:**

Many states have issued recommendations for the integration of SEL into schools. The proposed study uses a rigorous methodology to determine if the ACT OUT! psychodramatic intervention is a cost-effective means of bolstering SEC and reducing bullying incidence in schools.

**Trial Registration:**

ClinicalTrials.gov NCT04097496; https://clinicaltrials.gov/ct2/show/NCT04097496

**International Registered Report Identifier (IRRID):**

PRR1-10.2196/17900

## Introduction

### Social-Emotional Learning and Bullying

In the United States, students typically spend 1224 hours each year at school (6.8 hours per day for 180 days/year) [1], which is a substantial portion of their developmental lives. In this context, the past 25 years have seen renewed calls for the US education system to focus on holistic child development and social and emotional learning (SEL) in addition to standardized academic metrics [2]. SEL is widely considered to be important for improving students’ academic and nonacademic outcomes [3]. The implementation of SEL curricula, and even the core definition of SEL, does vary somewhat [4], and researchers continue to design and test SEL programs and implementation approaches in educational settings, typically using a framework advanced by the Collaborative for Academic, Social, and Emotional Learning (CASEL), which focuses on 5 domains: self-awareness, self-management, social awareness, relationship skills, and responsible decision making [5]. Learning in these domains is expected to foster corresponding social-emotional competence (SEC) [6]. A model from a recent study (*Conceptual Model for Advancing SEL in Schools*) by Greenberg et al [7] proposed that an overall interaction between SEL and SEC domains and program implementation leads to the improvement of various short-term and long-term outcomes for students. Although individual study results have varied considerably, an examination of 4 meta-analyses of hundreds of SEL studies in the US education system found substantive evidence for short-term benefits across a wide spectrum of outcomes, including targeted SEC outcomes, improved academic performance, reduced problematic conduct frequency, and lower emotional distress. Longer-term outcomes were significant in 2 meta-analyses for academic achievement [8].

Importantly, these positive outcomes appear in some cases to extend to serious school-related behavioral issues, such as bullying. Bullying is relatively ubiquitous in US schools. The prevalence of school bullying reported in a 2014 meta-analysis of 80 studies that included data on youth aged 12-18 years was 35% for traditional bullying and 15% for cyberbullying [9]. Thus, it is notable that several studies have observed an association between SEL programs and/or constructs and the attenuation of bullying or aggressive behavior [10-12], and an inverse relationship exists between perceptions that SEL instruction is being offered and perceptions of bullying at school [13].

Although best practices for SEL education have been proposed and are being refined, there remain many barriers to implementation. These include costs related to training and teachers’ time to learn about and teach SEL curricula, perceived inability to divert instructional time to SEL, potential lack of fidelity due to selective use of manualized elements, and competition with academic testing time [14,15]. For some SEL curricula, teacher time comprises a large fraction of the measured cost [16], although accurate measurement of costs and benefits of a given SEL curriculum is complex and subject to meaningful variability [17]. In addition, some scholars have been critical of the level of rigor of many SEL and SEC studies [18], asserting that the positive findings from meta-analyses may be overestimated or that the speed of SEL program adoption may exceed the generation of knowledge and understanding of how to maintain positive outcomes [19]. The current state of knowledge suggests the continued importance of research into SEL programming, both as an outcome (ie, development of SEC through SEL) and as a mediator of problem behavior (eg, bullying). To advance what is known, SEL research should prioritize methodological rigor and approaches that minimize school resource costs.

### The Proposed Study

This study will be an assessment of the ACT OUT! social issue theater program as a universal SEL intervention targeting SEC and bullying in elementary, middle, and high school students. ACT OUT! is an existing program that has been performed in various forms by professionally trained members of an acting ensemble since 1995 [20]. The present iteration consists of 3 distinct 15-min scenarios per grade range (elementary, middle, and high) that present age-appropriate improvisational dramas that illustrate issues related to SEL and bullying, including facilitated discussion between the actors—who remain in character—and the students. The program lasts approximately 1 hour to fit within a typical class period within the school day, including introductions, performance, and engagement.

ACT OUT! may be contrasted with typical SEL curricula. SEL curricula tend to consist of manualized, structured classroom or multicomponent programs involving multiple sessions over time. The median number of sessions within an SEL program in a meta-analysis of 213 SEL studies was 24 [21]. Being 1 hour in duration, ACT OUT! is substantially shorter and is performed by third party, professional actors, thus meeting the goal of reduced school resource costs for SEL programming but potentially raising concerns about if such a relatively brief dose could reasonably be expected to produce an effect. Underlying this study is a supposition that unique properties of a dramatic performance may specifically trigger SEL responses. In Aristotle’s *Poetics*, which is the first known work on dramatic theory, it is written that a dramatic tragedy (in the Aristotelian sense) is designed to arouse certain feelings, “wherewith to accomplish catharsis of…emotions” (as cited in an academic essay by Rosenstein [22] from a translation by Richard McKeon). This precise mechanism underlies the development of psychodrama as a psychotherapeutic intervention, as combined action and verbalization can present a situation that elicits an emotional response “freed from the restricting stereotyped residues of past experience” [23]. Recent studies and meta-analyses have examined psychodrama as a means of prevention and/or behavior change with generally positive findings [24-30]. Researchers have also found that youth are receptive to psychodramatic elements as part of a larger prevention curriculum [31]. However, no published studies have measured any outcomes of a single-session psychodramatic SEL experience.

This will be the first study to examine if a short dose of interactive psychodrama can affect SEC metrics and bullying experiences in schoolchildren in either the short (2 weeks) or medium (6 months) term. To respond to recent criticism of SEL studies, we have chosen to utilize the SPIRIT 2013 clinical trial guidelines in developing this protocol to promote rigor, reproducibility, and transparency [32].

## Methods

### Ethics Approval and Consent to Participate

This study was granted approval by the Indiana University institutional review board (IRB; #1908563296).

### Trial Design

**Figure 1:**
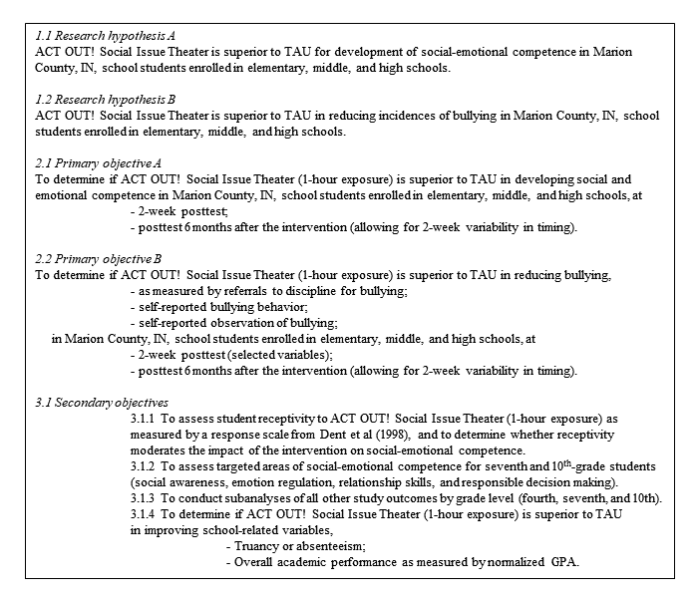
Hypotheses and objectives.

**Figure 2:**
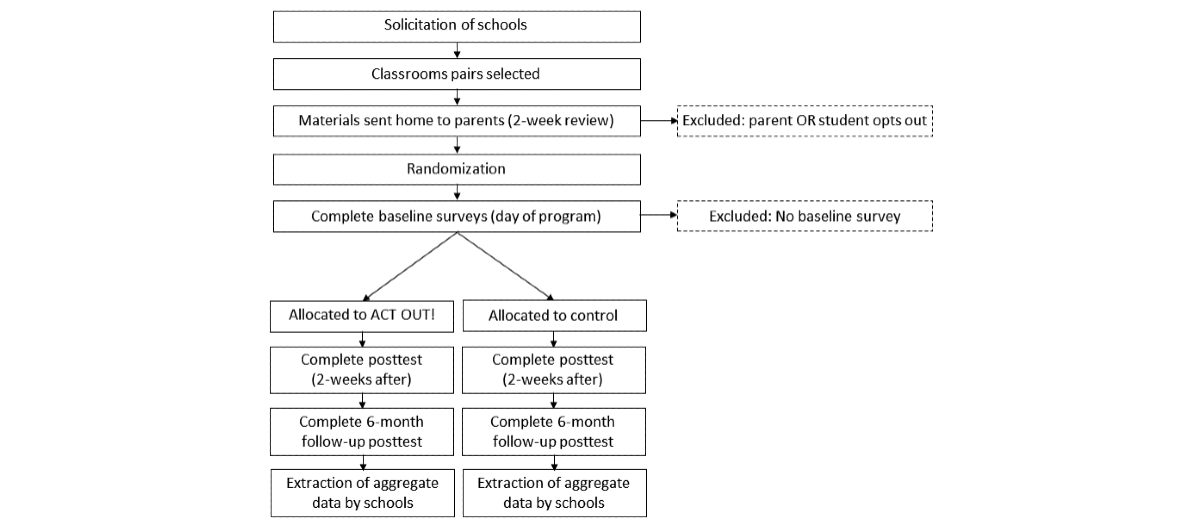
Participant timeline. GPA: grade point average; TAU: treatment as usual.

The ACT OUT! trial is designed as a proof-of-concept cluster randomized superiority trial with 2 parallel groups. Although the unit of measurement is the student, the unit of randomization is the classroom, stratified by school. For each grade (fourth, seventh, and 10th), an even number of classrooms will be selected from each school; half of the selected classrooms will be randomly assigned to the intervention arm and the other half will be assigned to the control arm. If there is an odd number of classrooms for a given grade and school, the excluded classroom will be determined randomly. The use of this approach will better ensure comparable sociodemographic and school-level factors between intervention and control arms. Study hypotheses and objectives are shown in Figure 1. Participant flow, including measurement points, is shown in Figure 2.

### Choice of Comparators

The comparator in this study is treatment as usual (TAU), which in this case reflects continued school activity as would have otherwise occurred. This study uses a novel and low-dose intervention in a protocol that would, in a clinical setting, approximate an early phase II stage. As a result, the core questions revolve around if the intervention works at all versus if it is superior to other mechanisms that may impact the same metrics. This is consistent with recent recommendations from an expert panel from the National Institutes of Health [33].

### Study Setting

The ACT OUT! trial will be conducted in public and charter schools in Marion County, Indiana, an urban county of 954,670 people who are 54.8% non-Hispanic white, 28.9% African American, and 10.6% Hispanic, with a median household income of US $44,689 [34]. The trial will recruit schools until meeting a threshold of 80 classrooms, 40 randomized to the intervention condition and 40 parallel classrooms randomized to the control condition.

### Eligibility Criteria

#### Inclusion Criteria

Participating classrooms must comprise fourth-grade (elementary), seventh-grade (middle), or 10th-grade (high) students. Single grades within each academic level were chosen to reduce variability that might be introduced by different age and maturity ranges when interpreting effects within levels. For elementary and middle school levels, the second-highest grade level was selected. For high school, 10th grade was selected because mandatory school attendance in Indiana ends at age 16, potentially introducing bias at the level of school enrollment for grades 11 and 12.

#### Exclusion Criteria

If a given grade within a school has an odd number of classrooms, 1 classroom will randomly be excluded from participation. Participants and their parents or legal guardians will review the study procedures. This study will utilize a waiver of active parental consent (see Recruitment section), and so parents or guardians may opt out on behalf of their dependents, and participants may opt out themselves.

### Intervention Delivery Qualifications

All individuals delivering the psychodrama interventions will be professional actors trained to the standards of Claude McNeal Productions, a professional theater company.

### Intervention

Eligible classrooms will be randomized to attend a 1-hour ACT OUT! interactive, semi-improvisational psychodrama performance or to continue with their school day as normal (TAU). Interventions will be delivered within the standard school day as defined for each classroom and school. The ACT OUT! production will include 3 vignettes paired with moderated discussions between the audience and the actors, and the latter will remain in character for the duration of the intervention. Intervention guidelines will be made available alongside the published study.

### Intervention Adherence

Adherence to the ACT OUT! intervention protocol will be measured at each delivery point using a fidelity checklist by a part-time employee of Claude McNeal Productions who is not a member of the acting ensemble. The fidelity checklist will contain scenario-specific items for all potential scenarios and will be made available as a multimedia appendix attached to the primary outcomes paper. Given the nature of improvisational psychodrama, the checklist will identify core areas and themes that must be addressed for the specific intervention to be considered to have been delivered with fidelity to the model. Each content area or procedure will be identified as *delivered/complete* or *undelivered/incomplete*, and the ratio will be used to generate a fidelity percentage for each delivery instance. To establish reliability, a second individual from the research team will attend 15% (6/40) of performances and complete fidelity checklists to compute interrater reliability (free-marginal kappa).

### Outcomes

#### Primary Outcomes

##### Overall Social-Emotional Competence at the 2-Week and 6-Month Posttests

SEC will be measured using the scaled variable generated by the Delaware Social-Emotional Competency Scale [35]. This scale reflects 4 of CASEL’s 5 core domains of SEC and has an established internal consistency coefficient of 0.84 in a large representative sample of youth. Furthermore, its developers demonstrated measurement invariance across grade levels, race, ethnicity, and gender [35]. This instrument provides an aggregate rating for overall SEC but cannot be used to generate separate scales by CASEL domains.

##### Self-Reported Bullying Behavior and Self-Reported Experiences of Being Bullied at the 2-Week and 6-Month Posttests

Bullying behavior and experiences of being bullied will be measured using the Bullying and Cyberbullying Scale for Adolescents (BCS-A), which uses 2 parallel 13-item scales [36]. This instrument includes items assessing physical, verbal, relational, and cyber bullying and was found in the development study to explain variance in adolescent problems beyond previously established tools—the Olweus Global Bullying Scale [37] and the Forms of Bullying Scale [38]—while displaying concurrent validity. The BCS-A also collects ratio rather than ordinal measures of bullying, which allows for face-valid use of the instrument for different time frames. As such, although the original BCS-A asked about a time frame in the past 3 months, this study will ask about a time frame in the past 2 weeks to match study protocols.

##### Referrals to Discipline for Bullying at the 6-Month Posttest

Referrals to discipline related to bullying will be obtained from school records in aggregate by cluster.

#### Secondary Outcomes

##### Student Receptivity to the ACT OUT! Intervention

This will be measured at the 2-week posttests using an instrument based on work published by Dent et al [39], which measures the degree to which adolescents find the event to be enjoyable, interesting, a waste of time, boring, understandable, difficult to understand, believable, important, and helpful, each on a 4-point ordinal scale.

##### Social-Emotional Competence Subdomains for Seventh- and 10th-Grade Students

The SEC subdomains of social awareness, emotion regulation, relationship skills, and responsible decision making will be measured using scales from the Washoe County School District Social-Emotional Competency Assessment [40]. These scales have established reliability and validity demonstrated through a unique multiyear, multimethod study, although the methods utilized did not produce traditional reliability values [40]. These items will not be collected from fourth-grade students because they would substantially increase the length of the survey instrument.

##### Truancy/Absenteeism and Academic Performance at 6-Months Postintervention

These will be assessed using objective data obtained from school records in aggregate by cluster.

### Sample Size

The sample size was calculated based on the primary outcome (change in SEC). Notably, there are no extant studies examining the effects of professionally acted interactive psychodrama on SEC outcomes and, hence, no directly applicable effect size estimates. However, several studies have utilized psychodrama/theater as an intervention component for non-SEC outcomes, including health promotion, sexual abuse prevention, HIV prevention, violence prevention, and nutrition education. Those studies found widely varying, significant effect sizes ranging from small to large, and, in cases where effect size calculations were not included, significant improvements in outcome metrics were found. The interventions varied substantially in quality, scope, and relationship with the measured outcomes [24-29]. An earlier Cochrane review covering the years 1990 to 2006 found only 9 studies of inconsistent quality, with generally modest but positive results [30]. Separately, a robust 2012 meta-analysis by Sklad et al [41] related to SEC identified 31 universal school-based programs that addressed social learning with an aggregated Cohen *d* of 0.70 (SE of *d* 0.10), a large effect size, but those programs were longer than the ACT OUT! intervention.

On the basis of this information, we have powered this trial to detect a moderate difference in SEC (Cohen *d*=0.30). For a baseline sample with a parallel superiority trial, with a 2-sided significance of .05, power of 0.80, and location of the mean in 1 group as a percentile of the other group set at 62% (corresponding to Cohen *d*=0.30), we calculated a need for 340 participants. As this is a cluster trial, we adjusted for similarities within the groups (clustering effect) using previous research on smoking prevention [42] as a conservative metric in the absence of similar data for SEL. This yielded an intracluster correlation (ICC) value of 0.153. The design effect, assuming 20 students per classroom (*m*), was calculated as [1+(m−1)×ICC], yielding 3.907. The design effect multiplied by the number of participants was 1328, but we also wanted to plan for approximately 20% loss to issues related to matching and attrition. Thus, we calculated a final sample need of 1594 students, constituting an estimated 80 classrooms evenly split into intervention and control arms.

### Recruitment

Each school involved in the ACT OUT! trial will be chosen based on its receptiveness and interest in participating in the project. Only schools that have provided a signed letter of agreement from an authorized official will be allowed to participate in the study. This study will utilize a waiver of parental consent (opt out), which was approved as part of the IRB submission. Neither the intervention nor the questionnaire poses more than minimal risk to participants or anyone else secondarily connected with the study (eg, their families and teachers). Surveys will be grouped by classroom and administered in a confidential manner. As part of this protocol, a notification letter and copy of the complete posttest survey instrument will be sent home to parents a minimum of 2 weeks before initial subject interaction (parent letter included as Multimedia Appendix 1). Parents and/or guardians will be able to opt their child out of the study simply by returning the second page of the letter to the school. Students will be provided with a brief summary of the study at the top of each survey form. They will be informed that they may also opt out of the study by simply not completing the instrument or by selectively skipping items based on comfort level.

The practical purpose of using a waiver of parental consent in recruitment is the substantial difference in participation prevalence as well as potential ethical concerns resulting from the probable exclusion of underrepresented minorities and high-risk populations, as in the study by White et al [43]. Indeed, several recent studies have suggested that active parental consent and opt-in methods produce smaller student samples with different characteristics, including some related to this study (such as bullying) [43-45]. These issues mirror similar findings related to sociodemographics, risk behaviors, and study participation reported in the previous several decades of school-based research [46-49]. In general, these studies imply that active parental consent may reduce sample size, introduce bias, and thus limit the generalizability of studies with youth.

### Assignment of Interventions

#### Allocation Sequence Generation

This trial is a cluster randomized trial; for each pair of classrooms (eg, 2 seventh-grade classrooms at the same school), a computerized random number generator will assign the classrooms to intervention (0) or control (1).

#### Allocation Concealment

The opt-out process will occur before the assignment. Allocation sequence will be concealed to the member of the research team who will assign each classroom to either the intervention or control group, until the moment of assignment.

#### Allocation Implementation

Allocation sequence generation will be completed by the research team, and data will be provided to participating schools after consent/assent has been finalized.

### Blinding

Due to the nature of the ACT OUT! trial, blinding of the trial participants, school officials, and members of Claude McNeal Productions is not possible. Furthermore, as data management will occur via the primary research team, group identity cannot realistically be masked. However, we have included 2 statistical/methodological consultants in the project team who will be asked to verify all analyses using masked group assignment.

### Participant Timeline

Recruitment for the study will begin in the fall of 2019, and interventions are expected to conclude in December 2019. A visual participant timeline is included in Figure 2.

### Data Collection Methods

#### Survey Collection Procedures

Each classroom will be assigned a random cluster ID with fixed leading values for grade level and arm (eg, 4C-12345678). Each classroom will be given a study packet that contains the appropriate number of survey and response forms, a manila envelope, a white envelope, and an administrator checklist, the latter of which is available as Multimedia Appendix 2. All survey forms and manila envelopes will also be imprinted with the appropriate cluster ID.

A classroom teacher will oversee survey administration after reviewing the administrator checklist. The voluntary and confidential nature of the survey will repeatedly be emphasized per the checklist. The administrator will record the number of individuals present in the classroom on the manila envelope (serving as a denominator for the cluster response rate). Absence for the 2-week postsurvey will not prevent a participant from completing the 6-month postsurvey. All surveys distributed to students will be placed by students into the manila envelope upon completion, even if left blank, which will allow calculation of the numerator of the cluster response rate. Survey forms that were never handed out at all (*extras*) will be placed into the white envelope by the administrator.

#### Quality Control

Data will be collected using a form designed in Scantron Design Expert and scanned directly into a database using an Insight 700c scanner (Scantron) to avoid data entry errors associated with manual transcription.

Intervention fidelity will be recorded at each instance by an employee of Claude McNeal Productions who is not a member of the ACT OUT! ensemble. This individual will use a form generated by the researchers that documents congruence between the observed performance and a checklist of planned elements (element present/element absent). To establish reliability, a second individual from Prevention Insights will attend 15% (6/40) of performances and complete fidelity checklists to compute interrater reliability (free-marginal kappa). The checklist will be published as a multimedia appendix to the study results.

#### Retention

To increase individual-level participation in the study, researchers will utilize an assent procedure approved by the appropriate IRB. As noted previously, this procedure, as opposed to active parental consent, is appropriate when the risks associated with the study are minimal. Participants may, however, withdraw from the study for any reason at any time, either via parental or participant request.

As hypotheses will be tested at 2 points in time, individuals who drop out of the study after providing data at the first posttest will not be excluded from hypothesis testing for proximal (2-week) outcomes.

#### Data Matching

To facilitate confidentiality, researchers will need to establish longitudinal linkages between surveys without collecting identifying information. This presents a risk of potential data loss. Meta-analytic research published within several months of trial protocol development asserts that there is no currently accepted best-practice standard for how to accomplish this most effectively in a way that preserves confidentiality and maximizes accurate matching of data between waves of collection [50]. However, these researchers found numerous benefits to accomplishing this goal using self-generated identification codes (SGICs) versus alternate methods, including true anonymity, improved response quality, utility, and maximal compliance with regulatory requirements. Researchers have also noted that SGICs are more effective when applied within smaller units or clusters [51], as is the case here.

The primary disadvantage of using an SGIC is that participants must accurately remember and report the same information [50]. The most common protocol used in longitudinal matching has been to use a combination of gender, race, and various snippets of personal information that are theoretically memorable (eg, middle initial, birth month, and mother’s initial), as in the study by Kearney et al [52]. However, although such a method is generally a secure way of matching surveys without identifying individual participants, it may cause concern among parents or youth [53].

For this protocol, we opted to generate an SGIC using procedures drawn from computer science and informatics literature related to password security questions. These questions are used by both children and adults to retrieve lost passwords and are intended to be both secure and memorable [54], meeting the needs of the SGIC as identified in the survey research literature. In Rabkin’s review [54] of security questions from banking institutions, questions designated as *weak* included those that are inapplicable to large segments of the target population (eg, a question about spouses), those that are not memorable (eg, last name of kindergarten teacher), and those that are ambiguous (eg, multiple truthful answers are possible, or a response is not static, such as *favorite food*) [54]. Later research on this topic also found that memorability of responses is greater for personal questions than for numbers and that memorability for all items declines over time, although high-success questions will experience minimal decline in successful recall, even after a year [55].

Using this evidence, the project will generate an SGIC with the following elements:

- Classroom ID
- Gender
- Race
- Ethnicity
- “How many *older* brothers and sisters do you have?” (0, 1, 2, 3 or more)
- “What color is your *backpack*? (if it is several colors, what color is it *mostly*?; black, red, green, blue, brown, purple, pink, a different color, I do not have a backpack)
- “What was the name of your *first pet*? (If you have never had a pet before, write the word “None” here;*handwritten entry*)
- Seventh and 10th grades only “What are the last two digits in your school locker combination? (for example, if your combination is 5-13-27, you would put 27)” (*digit entry*, I do not have a lock)

Computerized matching will occur within cluster ID. First, direct matches will be identified, and then 1-off matches (eg, all questions similar but 1) will be identified per well-established recommendations [50]. Any multiple matches (eg, more than 2 posttests matching a pretest SGIC) will be partitioned along with nonmatched data for manual inspection. Importantly, the *first pet* question includes handwritten entry, and so obvious similarities in handwriting can be used as a secondary matching tool.

### Analytic Methods

#### General Issues

Missing data may result from nonresponse to specific items or attrition following the baseline survey (eg, dropout following pretest but before any other analyses), and the type of missingness will be analyzed. Potential types include missing completely at random (MCAR; missingness is not related to the scores of any measured variable), missing at random (MAR; missingness is related to values of other measured variables, but not to the scores of that variable itself), or missing not at random (MNAR; missingness is related to scores of that variable itself). Data that are MCAR and MAR will be managed using multiple imputation [56]. As we expect some uncertainty in matching students across time points, each baseline survey will be considered a primary key, and surveys at postintervention will only be matched with each students’ baseline survey if researchers are reasonably certain that the ID code is a match from prespecified criteria. Surveys from postintervention that cannot be matched will not be included in the analysis. Students missing postintervention surveys will be considered missing due to attrition, and they will be included in the multiple imputation analysis as intention to treat.

Sensitivity analyses will be performed for plausible cases of data MNAR to examine the consistency of results across models. If the results are consistent, we will conclude that the conclusions are not compromised. We will also evaluate all appropriate statistical assumptions, such as outliers, variance heterogeneity, specification error, and nonnormality, before analysis.

#### Statistical Analysis

The intervention arm (ACT OUT! participation) will be compared against the control arm (TAU) in testing the primary hypotheses using an intention-to-treat model. This means that all individuals who are randomized will be included in the main analyses. As shown in Table 1, linear mixed models (LMM) or generalized LMM will be used to test all hypotheses. Baseline values will be included as covariates in each LMM, and all outcome values except receptivity and school-level data (truancy, absenteeism, and referrals to discipline for bullying) will be captured at baseline. Both fixed and random effects will be evaluated.

**Table 1:** Hypotheses, measures, and methods of analysis.

Outcome type and hypothesis		Time frame	Outcome measure	Method of analysis
**Primary**				
	Intervention improves short-term SEC^a^; long-term SEC	2-week follow-up; 6-month follow-up	DSECS-S^b^ (continuous)	LMM^c^; GLMM^d^ if nonnormal data
	Intervention reduces incidence of bullying others	2-week follow-up; 6-month follow-up	Thomas et al [36] 13-item bullying others scale (continuous)	LMM; GLMM if nonnormal data
	Intervention reduces incidence of being bullied	2-week follow-up; 6-month follow-up	Thomas et al [36] 13-item bullying scale (continuous)	LMM; GLMM if nonnormal data
	Intervention reduces incidence of referrals to discipline for bullying	6-month follow-up	Objective count data (continuous)	GLMM
**Secondary**				
	Intervention improves social awareness (seventh and 10th grades only)	2-week follow-up; 6-month follow-up	WCSD^e^ social-emotional competency assessment (continuous)	LMM; GLMM if nonnormal data
	Intervention improves emotion regulation (seventh and 10th grades only)	2-week follow-up; 6-month follow-up	WCSD social-emotional competency assessment (continuous)	LMM; GLMM if nonnormal data
	Intervention improves relationship skills (seventh and 10th grades only)	2-week follow-up; 6-month follow-up	WCSD social-emotional competency assessment (continuous)	LMM; GLMM if nonormal data
	Intervention improves responsible decision making (seventh and 10th grades only)	2-week follow-up; 6-month follow-up	WCSD social-emotional competency assessment (continuous)	LMM; GLMM if nonnormal data
	Receptivity to ACT OUT! will moderate impact of the intervention	2-week follow-up; 6-month follow-up	Dent et al [39] instrument (continuous)	LMM; GLMM if nonnormal data
	Explore outcome differences by grade level (fourth, seventh, and 10th grades)	2-week follow-up; 6-month follow-up	Four outcome measures from primary objectives (all continuous)	LMM; GLMM if nonnormal data
	Intervention will improve truancy and absenteeism	6-month follow-up	Objective count data (continuous)	GLMM
	Intervention will improve academic performance	6-month follow-up	GPA^f^ standardized to a 4.0 scale (continuous)	LMM; GLMM if nonnormal data

^a^SEC: social-emotional competence.

^b^DSECS-S: Delaware Social-Emotional Competency Scale.

^c^LMM: linear mixed model.

^d^GLMM: generalized linear mixed model.

^e^WCSD: Washoe County School District.

^f^GPA: grade point average.

## Results

This study was funded in June 2019; approved by the Indiana University IRB on September 17, 2019; began subject recruitment and data collection on November 5, 2019; and prospectively registered with ClinicalTrials.gov. This protocol paper, except this paragraph, was submitted as prepared in September 2019 to maintain transparency regarding changes between the proposed protocol and the finished study.

## Discussion

### Significance of the Study

SEL, SEC, and bullying potentially affect all schoolchildren; in recognition of this, states such as Indiana have convened bodies (eg, Indiana Commission on Improving the Status of Children in Indiana) and issued recommendations for the integration of SEL into schools [57]. At the same time, many barriers to effective program implementation remain [14-16], and the rigor of existing evaluation studies has been questioned [18]. Given the importance of these issues, this proposed study tests a novel, cost-effective intervention structure using a rigorous methodology, including this study protocol, which has been completed and registered before any subject enrollment.

### Data Monitoring, Interim Analyses, and Auditing

This study will not have a data monitoring committee because the anticipated risks are minimal, and the active duration of the intervention is short within each cluster. Harms will nonetheless be evaluated in the highly unlikely event that they accrue in a manner attributable to this intervention. In addition, no interim analyses are planned other than following the 2 planned analytical timepoints indicated in Table 1. Data and analyses will be audited by multiple project consultants who are members of the project team but who are not part of Prevention Insights, the unit directly funded to support Claude McNeal Productions.

### Limitations

The proposed study has several potential limitations. The protocol is designed to test outcomes from a low-dose intervention, which, being 1 hour in length, fits within a standard class period and does not unduly burden school personnel. However, it is likely that multiple doses would provide a more robust effect (if an effect exists), and so, the pragmatic decision to test a single iteration of ACT OUT! for this study may contribute to a potential type II error. Furthermore, it is possible that an unexpectedly large percentage of students will be unable to be matched over time, especially at the 6-month posttest, due to the confidentiality requirements of the study. Use of an SGIC is a best-practice matching technique, but results from studies using this technique have disagreed about how to operationalize it and have produced a fairly wide range of matched percentages. However, collection of identifying data would require active parental consent, which itself has been shown to substantially reduce participation rates and to bias samples in ways that could meaningfully affect this study. Hence, use of the confidential SGIC to enable assent procedures is likely more robust than an identified matching protocol. Finally, it is possible that the treatment and control classes will display cross contamination, in which control subjects are informed by intervention subjects about the program. This can be identified at follow-up, and, more importantly, given that the emotional response to ACT OUT! is hypothesized to be an important mechanism of change, this threat is likely minimal because the control subjects will not have been exposed to the actual ACT OUT! experience.

## Appendix

Multimedia Appendix 1 Sample parent notification letter.

Multimedia Appendix 2 Classroom administration checklist.

## Abbreviations

BCS-A: Bullying and Cyberbullying Scale for Adolescents
CASEL: Collaborative for Academic, Social, and Emotional Learning
ICC: intracluster correlation
IRB: institutional review board
LMM: linear mixed models
MAR: missing at random
MCAR: missing completely at random
MNAR: missing not at random
SEC: social-emotional competence
SEL: social and emotional learning
SGIC: self-generated identification code
TAU: treatment as usual
