# Supplementary material for: Effects of the ACT OUT! Social Issue Theater Program on Social-Emotional Competence and Bullying in Youth and Adolescents: Protocol for a Cluster Randomized Controlled Trial
Source: JMIR Res Protoc. 2020 Apr 13;9(4):e17900. doi: 10.2196/17900 (PMC7186869; doi:10.2196/17900)
Supplement: Multimedia Appendix 1 [file resprot_v9i4e17900_app1.docx]

**ACT OUT! Study Parent Notification Letter**

Your child is being asked to participate in a voluntary study of the *Act Out!* theater program (<https://www.claudemcnealproductions.com/act-out-ensemble/>). The purpose of the study is to learn whether watching the 1-hour *Act Out!* performance helps children develop social and emotional skills (e.g., “I think about how others feel”) and engage in fewer bullying behaviors (e.g., “I/we spread false rumors about a person, to hurt them or make others not like them”).

**Why is your child being asked to participate?**
Claude McNeal Productions has delivered variations of the *Act Out!* social issue theater program in elementary, middle, and high schools for more than 20 years, reaching nearly 500,000 individuals. Leaders at Claude McNeal Productions would like to learn more about how Act Out! benefits youth. *Prevention Insights* at the Indiana University School of Public Health is assisting with designing and conducting a research study to determine whether the *Act Out!* program produces benefits in the areas of social/emotional competence and bullying reduction.

Your child has been asked to participate in the study because he or she is a student in 4^th^, 7^th^, or 10^th^ grade whose school expressed interest in receiving free performances of *Act Out!* Your child’s participation in this study is voluntary. Your and your child’s decision about whether or not to participate will not affect anyone’s future current or future dealings with your child’s school, Indiana University or Claude McNeal Productions. If you decide your child can participate, you or your child may still withdraw at any time in the future without affecting those relationships.

**What procedures are involved?**Participation in the study will involve classrooms being randomly assigned to either watch *Act Out!* or continue with their school day as usual. Half of the classrooms will view *Act Out!* and half will continue their school day as normal. Regardless of whether your child’s classroom sees the performance or not, your child will be asked to complete a short survey about social-emotional learning and bullying. The survey will be confidentially administered (no names) by a classroom teacher or community member who has been trained to administer the survey. The survey will be administered three times over the course of a full school year and should take fewer than 25 minutes each time.

**What are the potential risks?**Risks related to participating are minimal, but some questions do ask about potentially sensitive issues related to bullying that might make some students feel uncomfortable.

**What are the potential benefits?**The benefits to this research include helping learn whether the *Act Out!* program is a beneficial investment for schools that are interested in enhancing youth development and reducing bullying.

**What other options are there?**
You have the option to refuse your child’s participation in the surveys, and/or your child’s viewing of the *Act Out!* program. If you do not want your child to participate, he or she will be asked to participate in an alternate activity, such as reading in the classroom, while the survey and/or program are taking place.

Your child may also withdraw from the study at any time and for any reason.

**Will my child’s study-related information be kept confidential?**
We will use all reasonable efforts to ensure that the survey is confidential. Because we are interested in understanding changes in social and emotional skills, we are asking several questions that we hope can link the surveys confidentially (e.g., knowing which pairs of surveys go together without knowing the identity of the person who took any of them).

4^th^ Grade: Name of first pet, color of backpack

7^th^ and 10^th^ Grades: Name of first pet, color of backpack, last number in their locker combination at school

We understand that we will not be able to link all surveys using this method but chose to err on the side of caution (e.g., not using middle initials or date of birth) to maximize the confidential nature of the study.

**What if I don’t want my child to participate?**
Simply fill out the form on the next page and have your child return it to his or her homeroom teacher. You do not need to complete this form if you are allowing your child to participate.

**Questions**For questions about your rights as a research participant, to discuss problems, complaints, or concerns about a research study, or to obtain information or to offer input, please contact the IU Human Subjects Office at 800-696-2949 or at irb@iu.edu.

**Principal Investigator Name:** Dr. Jon Agley, Associate Professor and Deputy Director of Research

**Department and Institution:** Prevention Insights, School of Public Health, Indiana University, Bloomington

**Contact Information:** 812-855-3123

**Grant Support:** Lilly Endowment Inc.

**ACT OUT! Study Opt-Out Form**

You **only** need to complete this form if you **do not** want your child to participate in the act out study. If you do not want your child to participate, return this form with your signature to your child’s homeroom teacher.

_______ I do **not** want my child to participate in the *Act Out!* study.

Parent/Guardian Signature ____________________________________________

Parent/Guardian Written Name _________________________________________

Child’s Name _______________________________________________________

Homeroom Teacher __________________________________________________
