# Supplementary material for: Effects of the ACT OUT! Social Issue Theater Program on Social-Emotional Competence and Bullying in Youth and Adolescents: Protocol for a Cluster Randomized Controlled Trial
Source: JMIR Res Protoc. 2020 Apr 13;9(4):e17900. doi: 10.2196/17900 (PMC7186869; doi:10.2196/17900)
Supplement: Multimedia Appendix 2 [file resprot_v9i4e17900_app2.docx]

____ Have the room set up as if you are administering an exam/test – lots of space between students to the extent reasonable/possible.

____ Please have extra #2 lead pencils available for students.

____ Record the number of students in the group on the manila envelope. **Do not write down any other information (especially if it might identify the group).**

____ Distribute one survey form and answer sheet to each student. Use only originals; no photocopies. There are two types of survey forms, one for **4^th^** graders and the other for **7^th^ and 10^th^** graders. Make sure that you have the right forms.

____ Place the manila envelope in the front of the room (where it can be accessed easily, but not directly in front of you) and instruct the students to place their **survey forms** in the **manila** envelope (even if they choose to leave the survey form blank).

____ Place **extra** survey forms (i.e., those that were NOT distributed) in the **white** envelope. Keep **unused** survey forms separate from the distributed survey forms.

____ Review the following instructions with your students:

- Participation is voluntary and all responses are confidential.
- Please answer the questions truthfully; no response will be matched to a particular student.
- Please only mark on the answer sheet.
- Students may leave any or all questions blank. It is better to leave a question blank than to answer it randomly.
- Students should place both forms **(even if they decided to leave it blank)** in the manila envelope. Every survey form that was handed out should end up in the manila envelope, no matter if the student filled it out or not.
- If this is the pre-test survey, and your classroom has been randomly selected to attend Act Out!, you **must** inform students that they are not required to attend the performance, and may instead participate in a quiet activity in the classroom or other school location.

**(Over)**

____ Administrator should remain seated at his/her desk for the duration of the survey to assure confidentiality.

____ When all students have placed their survey forms in the manila envelope, seal the envelope. Please **do not sort or straighten** the completed survey forms.

____ Return the sealed manila envelope (the distributed survey forms) and the white envelope (the non-distributed survey forms) to the Survey Coordinator as soon as possible.
